# Bodily Distress Syndrome Is Associated with Impaired Physical Fitness—A Population Based Cross-Sectional Study (DanFunD)

**DOI:** 10.3390/jcm13216373

**Published:** 2024-10-24

**Authors:** Rebecca Dalby Bavnhøj, Anne Ahrendt Bjerregaard, Anja Lykke Madsen, Signe Ulfbeck Schovsbo, Marie Weinreich Petersen, Per Fink, Matilde Winther-Jensen, Torben Jørgensen, Line Lund Kårhus, Thomas Meinertz Dantoft

**Affiliations:** 1 Center for Clinical Research and Prevention, Copenhagen University Hospital—Bispebjerg and Frederiksberg, 2000 Frederiksberg, Denmark; rebecca.dalby@hotmail.com (R.D.B.); anne.ahrendt.bjerregaard@regionh.dk (A.A.B.); anja.lykke.madsen@regionh.dk (A.L.M.); signe.ulfbeck.schovsbo@regionh.dk (S.U.S.); matilde.winther-jensen.01@regionh.dk (M.W.-J.); torben.joergensen@regionh.dk (T.J.); line.lund.kaarhus@regionh.dk (L.L.K.); 2 Research Clinic for Functional Disorders and Psychosomatics, Aarhus University Hospital, 8200 Aarhus, Denmark; marie.weinreich.petersen@auh.rm.dk (M.W.P.); per.fink@aarhus.rm.dk (P.F.); 3 Department of Clinical Medicine, Aarhus University, 8000 Aarhus, Denmark; 4 Department of Public Health, Faculty of Health and Medical Science, University of Copenhagen, 1172 Copenhagen, Denmark

**Keywords:** functional somatic disorder, DanFunD, bodily distress syndrome, cardiorespiratory health, physical performance, body composition

## Abstract

**Background and Aim:** Functional somatic disorders (FSDs) are a unifying diagnosis that includes functional somatic syndromes (FSSs) as well as the unifying diagnostic construct of bodily distress syndrome (BDS). FSDs are characterized by persistent and troublesome physical symptoms that are prevalent across all medical settings and for which no clinical tests can establish a definitive diagnosis. The aim of this study was to explore associations between BDSs and objective measurements of body composition, cardiorespiratory health, and physical performance. **Methods:** Analyses are based on data from the Danish population-based cohort study, DanFunD, comprising data on 9656 participants aged 18–76 years and BDS case status, which was established using self-reported questionnaires. Adjusted multiple linear regression analysis was employed to evaluate associations between BDS and different measures of body composition, cardiorespiratory health, and physical performance assessed as part of a general health examination. **Results:** Compared to controls, individuals with single- or multi-organ BDS exhibited less optimal body compositions characterized by a higher BMI and fat percentage and larger waist circumference, as well as impaired cardiorespiratory health and reduced physical performance (lower maximal oxygen consumption and lower hand grip strength). Further, individuals categorized with multi-organ BDS had a less healthy body composition, lower cardiorespiratory health, and lower physical performance compared to individuals with single-organ BDS. **Conclusions:** In this cross-sectional study, we found BDS to be associated with suboptimal body composition, impaired cardiorespiratory health, and reduced physical performance. Individuals with multi-organ BDS tended to exhibit lower physical fitness or reduced cardiorespiratory health than individuals with single-organ BDS.

## 1. Introduction

Functional Somatic Disorders (FSDs) are characterized by persistent and troublesome physical symptoms that cannot be attributed to a known physical or mental condition and for which no clinical tests can establish a diagnosis [1,2]. FSDs are also referred to in the literature by alternative labels, such as medically unexplained symptoms, somatoform disorder, and somatic symptom disorder [3,4,5]. Our current understanding of FSDs regarding etiology, pathogenesis, and risk factors is still at an early stage, but the etiology is considered to be multifactorial, with social, psychological, and biological factors being involved. The symptoms reported vary from patient to patient and may appear from different bodily systems [1]. Previous studies have found that the most frequent symptoms include general exhaustion and pain in the musculoskeletal system, such as backache, joint pain, and muscle pain [6,7]. The severity of FSD also varies, ranging from being milder to more severely disabling. Severe cases of FSD are associated with excessive use of healthcare services and a lower self-perceived social status [8,9].

Some examples of FSD include functional somatic syndromes (FSSs), such as irritable bowel syndrome, fibromyalgia, and chronic fatigue syndrome, as well as the diagnostic concept of bodily distress syndrome (BDS) [10,11]. Given that the symptoms of FSS share documented symptomatic overlap, the BDS construct was developed as a unifying delimitation of FSD. In contrast to the FSS, BDS encompasses symptoms from multiple bodily systems within a single diagnosis [10]. The BDS construct is well-validated and has been shown to encompass a range of FSSs [12]. Previous questionnaire-based studies have estimated the prevalence of FSD (defined according to the BDS criteria) in the Danish population at around 15–16%, and thus, FSD is a frequently diagnosed condition [6,11].

Although FSD is characterized by persistent and troublesome physical symptoms, studies on cardiorespiratory health and physical performance in individuals with FSD are limited. Nevertheless, associations between BDS and lower self-rated mental health, decreased physical health, and being overweight and obese have been reported [6]. Correspondingly, BDS has also been found to be associated with more frequent use of opioids and medical services, higher risk of work disability, and higher health care costs, indicative of more severe physical symptoms [6]. Lower levels of physical activity have been linked to poorer long-term health status [13]. If patients with FSD more commonly experience poor physiological health, counseling and other health promotion initiatives could positively impact their overall well-being and potentially reduce the severity of their symptoms. Therefore, the aim of this study was to investigate associations between BDS and objective measurements of body composition, cardiorespiratory health, and physical performance. It was hypothesized that cases with BDS would exhibit lower physical fitness and reduced cardiorespiratory health than controls with no BDS and that those with multi-organ BDS would show a more pronounced impairment compared to those with single-organ BDS.

## 2. Material and Methods

### 2.1. Study Population

This present study is based on data from the Danish Study of Functional Disorders (DanFunD) and is a general population-based cohort study with the superior aim of studying the epidemiology of FSD. The cohort has been described in more detail elsewhere [14]. Briefly, the cohort consisted of 9656 (33.7%) randomly invited men and women aged 18–76 years and residing in the western part of the greater Copenhagen, Denmark. Participants were randomly obtained from the Danish National Civil Registration system, and exclusion criteria were not having been born in Denmark, non-Danish citizens, and pregnancy. All participants completed both a general health examination conducted by trained and experienced research nurses and filled in self-administered paper-based questionnaires. Written informed consent was obtained from all participants before participation, and this study received approval from the Ethical Committee of Copenhagen County (Ethics Committee: H-3-2011-081, and H-3-2012-0015) and the Danish Data Protection Agency [14].

### 2.2. Case Definitions of BDS

Cases with BDS were identified from ‘The BDS checklist’, which is a questionnaire covering bothersome symptoms within the last 12 months. The BDS checklist includes 25 symptoms distributed across four symptom clusters: cardiopulmonary (*CP)* symptoms, gastrointestinal (*GI)* symptoms, musculoskeletal (*MS)* symptoms, and general (*GS)* symptoms. Each symptom is rated on a five-point Likert Scale ranging from ‘not at all’ to ‘a bit’, ‘somewhat’, ‘quite a bit’, and ‘a lot’ bothered by symptoms within the last 12 months. Participants who reported being bothered ‘somewhat’, ‘quite a bit’, or ‘a lot’ were considered as bothered by the symptom. BDS was assigned to participants bothered by at least four symptoms within at least one of the four BDS symptom clusters. BDS divides individuals into a single/oligo-organ subtype, encompassing persons with symptoms from one or two of the four symptom subgroups, and a multi-organ subtype, which includes those with at least four symptoms from at least three of the four symptom subgroups [11,12]. Participants with missing data on BDS status were excluded from analyses. Otherwise, no other exclusion criteria were applied.

### 2.3. Outcome Assessment

All outcomes were assessed during the health examination conducted at the Research Centre for Prevention and Health (now Centre for Clinical Research and Prevention) in Copenhagen between 11 November 2011 and 30 June 2015. Standardized and detailed research protocols were prepared for each outcome measure following WHO-recommended procedures when available or according to the manufacturer’s instructions. In addition, reproducibility among staff and across the study period was ensured through periodical internal validations. Details of each outcome and its specific measurement are described below.

#### 2.3.1. Body Composition

*Waist circumference* was measured in centimeters (cm) by placing the measuring tape midway between the lower rib curvature and the upper edge of the hip of the study participant. The World Health Organization cut-off values were used to estimate the proportion of individuals exceeding the optimal waist circumference, which is 102 cm for men and 88 cm for women [15]. *Body fat percentage* (%) was measured once using a Tanita TBF-300 body composition analyzer (TANITA, Middlesex, UK). Body mass index (BMI) (kg/m^2^) was calculated based on weight and height without shoes and categorized into three nutritional classifications according to the definitions in ICD-11, i.e., underweight or normal weight: BMI of 24.9 or below; overweight: BMI between 25.0–29.9; obese (class I–III): BMI equal to or greater than 30.0 [16].

#### 2.3.2. Cardiorespiratory Health and Physical Performance

*Systolic and diastolic blood pressure (mmHg)* were measured with a mercury manometer (Speidel & Keller, Jungingen, Germany) with the participant in a sitting position and after five minutes of rest. Two measurements were taken one minute apart, and the mean of the two measurements was calculated and used in the analyses.

*Pulmonary function* was assessed by spirometry with the participant in a sitting position (Spiropharma A/S, Poland). Pulmonary function was measured in three different ways. Forced Vital Capacity in the first second (FVC, L) measures the total amount of air a person can forcefully exhale from the lungs. Forced Expiratory Volume in the first second (FEV_1_, L) is a measure of the amount of air one person can forcefully exhale from the lungs in the first second. Further, the FEV_1_/FVC ratio was calculated to evaluate obstructive lung conditions. At least three measurements were performed for each participant, and the highest values of FVC and FEV1 were recorded.

*Maximal oxygen consumption* (VO_2_-max) was estimated using a step bench with variable height (20–35 cm) (The original step, Hicksville, NY, USA) dependent on the sex and fitness level of the participant. The VO_2_-max was estimated from the time that the participant could step on the step bench in a slowly increasing rhythm [17].

A hand-grip test was performed using a dynamometer (Jamar, Duluth, MN, USA), with three repetitions in both the right and left hand. The dominant hand was recorded, the average of the two first repetitions was used in analyses, and max strength was estimated in kilograms of force applied [18].

#### 2.3.3. Covariates

*Dietary intake* was estimated using a self-administered 26-item food frequency questionnaire [FFQ] classified into three categories (healthy, neutral, unhealthy) [19]. *Smoking habits* were assessed with a single item asking if the participant was a “daily smoker”, “occasional smoker”, “former smoker”, or “never smoker”. Responses were dichotomized into either daily/regularly or former/never.

*Alcohol intake* was assessed as the number of drinks (13 g of alcohol each) per week [20].

*Daily physical activity* was assessed using the “Saltin-Grimby Physical Activity Level Scale” designed to estimate the level of physical activity during leisure time. The responses were dichotomized into sedentary/some physical activity or regular physical activity/regular hard physical training [21].

*Sleep disturbances* were measured with a single question on a 5-point Likert scale, which was dichotomized into daily/almost daily or weekly/monthly/rarely.

*Self-perceived stress* was assessed using the 10-item version of Cohens’s Perceived Stress scale (PPS). The PSS estimates the degree to which situations in one’s life are perceived as stressful and provides a global measure of perceived stress over the previous month. Each question was scored on a 5-point Likert scale ranging from “0 = never” as the lowest to “5 = very often” as the highest and evaluated with a final score ranging from zero to 40, where higher scores indicate a higher level of self-perceived stress [22].

*Anxiety and depression* were assessed using eight items from the Anxiety and Depression subscales from the 90-item Symptom Checklist 90 (SCL-90) [23,24]. Each question was scored on a 5-point rating scale ranging from “0 = not at all” as the lowest score to “5 = a great deal” as the highest. The total sum score ranged from 0 to 32, and, for both subscales, higher scores reflect a higher level of anxiety and depression, respectively.

## 3. Statistical Analyses

Statistical analyses were performed using SAS Enterprise Guide 8.3, and results were evaluated at a 5% significance level. Standard descriptive statistics (*n* (%)), mean and standard deviation, median, and 25 and 75 percentiles (q1–q3) were applied to describe the distributions of outcomes and covariates within overall BDS, single organ-BDS, multi-organ BDS, and controls, respectively. To test the difference between overall BDS, single-organ BDS, multi-organ BDS, and controls, a *t*-test was applied for normally distributed continuous variables, the Kruskal–Wallis test was used for continuous variables with a skewed distribution, and a Chi-squared (X^2^) test was applied for categorical variables. Multiple linear regression analysis was performed using the SAS Genmod procedure to estimate associations between overall BDS, single-organ BDS, and multi-organ BDS as independent variables and body composition, cardiorespiratory health, and physical performance as dependent variables. Potential confounders included in the analyses were identified a priori based on a literature review directed acyclic graphs (DAGSs). To assess these associations, we included three progressively adjusted models to explore the robustness of the associations and to minimize the potential for confounding factors while addressing concerns of overfitting due to the number of independent variables. In model 1, adjustment was made for age and sex; model 2 was additionally adjusted for lifestyle factors (sleep, dietary habits, smoking, alcohol, and physical activity), and model 3 was further adjusted for mental health factors (self-perceived stress, anxiety, and depression). Lung function (FVC and FEV1) was additionally adjusted for weight and height in all models. Multicollinearity among the predictor variables was assessed using the Variance Inflation Factor (VIF) in the regression analysis. All predictor variables demonstrated acceptable levels of multicollinearity, with VIF values below 4, indicating no significant multicollinearity concerns in the model. Associations are reported as regression coefficients and 95% confidence intervals (CIs).

To test whether the model assumptions were met, the residuals were checked for normally distributed and homogeneity of variance. If the model assumptions were not met, the outcome was log-transformed using the base 10 logarithm. The results presented were transformed back by calculating 10 to the power of the regression coefficients. A test of the linearity assumption showed that including age squared did improve the model fit slightly; however, the improvement was minimal.

## 4. Results

Out of the total population, 1544 participants (16.0%) fulfilled the criteria for BDS. Among them, 1448 individuals fulfilled the criteria for single-organ BDS (15.1% of the total population) and 96 (1% of the total population) for multi-organ BDS. A total of 8035 participants did not fulfill the criteria for BDS and were regarded as controls. A total of 77 participants (0.8% of the total population) had missing information about their BDS status and were excluded from the analysis.

Population characteristics are presented in Table 1. Participants with overall BDS, single-organ BDS, or multi-organ BDS were more often women. The median age was significantly lower among participants with multi-organ BDS and higher in single-organ BDS compared with the control population. Overall BDS, single-organ BDS, and multi-organ BDS patients exhibited a less optimal body composition (higher waist circumference, higher fat percentage, and higher BMI), as well as lower lung capacity (decreased FVC and FEV1), lower hand grip strength, and lower fitness capacity (decreased VO_2_-max) than the controls. Furthermore, overall BDS, single-organ BDS, and multi-organ BDS were associated with unhealthy diet, sleep disturbance, decreased mental health, and lower levels of daily physical activity compared with the controls. Cases with overall BDS and single-organ BDS were also more often daily or frequent tobacco smokers than the controls, whereas women with overall BDS or single-organ BDS were drinking significantly less alcohol than female controls. Among cases in multi-organ BDS, both men and women were drinking significantly less alcohol than the controls.

The regression analyses showed an association between higher waist circumference, higher fat percentage, and increased BMI with overall BDS, single-organ BDS, and multi-organ BDS compared with the controls, independent of age and sex, as well as lifestyle and mental health factors (Table 2). As for cardiorespiratory fitness and physical performance, overall BDS was negatively associated with FVC in the fully adjusted model, whereas, for single-organ BDS, the results were negatively associated with FCV after adjustment of sex, age, and lifestyle factors (models 1 and 2), but no longer after adjusting for mental health factors (Table 3). No significant associations were observed between multi-organ BDS and FVC or between overall BDS, single-organ BDS, or multi-organ BDS and FEV1, FEV1/FVC ratio, systolic blood pressure, or diastolic blood pressure, respectively (Table 3).

Significantly lower handgrip strength was found among cases in both overall BDS, single-organ BDS, and multi-organ BDS compared with the controls. Additionally, compared to the controls, overall BDS, single-organ BDS, and multi-organ BDS were found to be associated with a significantly lower VO_2_ max uptake (Table 3). Notable, approximately 14.3% of participants were unable to complete the step test, that is, n = 425 (27.5%) for overall BDS, n = 384 (26.5%) for single-organ BDS, n = 40 (41.7%) for multi-organ BDS and n = 943 (11.7%) for the control population. For all other measured outcomes, the number of missing values was minimal. Lower VO_2_ max uptake was found among cases in both overall BDS, single-organ BDS, and multi-organ BDS (Table 3). Approximately 14.3% of participants were unable to complete the step test, that is, n = 425 (27.5%) for overall BDS, n = 384 (26.5%) for single-organ BDS, n = 40 (41.7%) for multi-organ BDS, and n = 943 (11.7%) for the control population. For all other measured outcomes, the number of missing values was minimal.

## 5. Discussion

This study provides new insights into body composition, cardiorespiratory health, and physical performance among individuals with BDS, identified by screening a large randomly invited sample from the general adult population. Prior research on FSD and similar outcome measures has predominantly been conducted in clinical settings, where the phenotypic characteristics of study populations may differ significantly from those in a general population-based BDS sample.

In the present study, we found less optimal body composition to be associated with BDS, i.e., higher waist circumference, higher fat percentage, and higher BMI. Furthermore, although blood pressure did not differ between cases with BDS compared to the general population, we did find indications of impaired cardiorespiratory health and poorer physical performance associated with BDS, i.e., decreased lung function associated with overall BDS, as well as lower VO_2_ max and reduced muscle strength associated with both overall BDS and the two BDS subgroups. Since the ratio of FVC/FEV1 does not differ between BDS and control populations, the decreased lung function does not have an obstructive character [26]. The findings from this study are in line with previous reports of FSD being associated with both poor self-perceived health and limitations in everyday life [6,11].

Studies of body composition and FSD are limited, but the findings from the available data are comparable to ours. A longitudinal health study from Norway of nearly 16,000 randomly invited females found that high BMI (overweight and obese) was an independent risk factor for the future development of fibromyalgia [27]. Similarly, a Canadian community health survey with nearly 60,000 participants found that an unhealthy lifestyle, including obesity and smoking, was associated with both chronic fatigue syndrome and fibromyalgia [28]. Their findings are supported by findings of high rates of obesity in patient samples with IBS from Canada and Iraq [29,30]. In two previous studies, one of them also based on data from the DanFunD cohort, multiple chemical sensitivity, a condition also considered to be an FSS, was found to be associated with less optimal body composition, as well as lower lung function performance, impaired cardiorespiratory fitness, and lower physical performance [31,32]. No other studies have previously characterized cardiorespiratory health or physical performance in participants with BDS in a non-clinical sample. However, the findings of a lower fitness capacity (lower or no estimate of VO_2_-max) and lower hand grip strength among participants with BDS are supported by earlier reports of poorer self-perceived physical health and impaired self-perceived functional capacity among BDS individuals compared with controls [6,11]. Furthermore, in a clinical sample of patients with fibromyalgia from Turkey, Köklü and colleagues found that decreased handgrip strength and increased BMI were associated with fibromyalgia compared with healthy subjects [33]. Likewise, in a study comparing 272 patients with a medically confirmed myalgic encephalomyelitis/chronic fatigue syndrome (ME/CFS) diagnosis from the UK National Health Service with a healthy control group of 136 people with apparently normal function and no symptoms of fatigue nor any severe disease, ME/CFS was found to be associated with lower handgrip strength [34].

It is generally difficult to estimate the clinical significance of findings being based on a general population-based sample. However, the differences observed between cases with BDS and healthy controls in this study are of a magnitude of potential clinical relevance. For example, more than 40% of the multi-organ BDS cases and 24% of the single-organ BDS cases are obese, compared to only 15.5% in the non-BDS general population sample. Similar large differences are observed for waist circumference and also, to some degree, for body fat percentages. Differences observed for fitness, muscle strength, and lung functioning capacity are at the same negative magnitude and are also considerably worse for those with multi-organ BDS. In this regard, it is important to emphasize that participants with BDS have not been compared to a selected healthy population but to a random sample of the general adult population not fulfilling the criteria for BDS. Those findings suggest that an increased focus on health promotion among patients with BDS could positively impact their physical health and improve their quality of life.

The effectiveness of such an initiative will naturally depend on the severity of the disease and the physical and mental capacity of individual patients. Ultimately, it might also reduce the risk of comorbid chronic diseases, where factors like smoking, high BMI, and physical inactivity are well-known risk factors [25]. It is essential, of course, that the effects and consequences of such initiatives be examined in prospective studies and evaluated in clinical settings before they can be incorporated into clinical guidelines for FSD.

## 6. Strengths and Limitations

An important strength of this study is the large, random sample of nearly 10,000 participants from the DanFunD cohort, recruited from the general adult population. Another strength is that the health examinations were conducted by experienced research nurses following detailed and optimized protocols and that reproducibility between staff and across the study period was monitored by periodical internal validations by co-workers. Further, FSD case status in this present study is based on self-reported symptoms from the “BDS-Checklist”, which has been validated as a feasible screening tool for studying FSD [10].

A possible limitation of this study is the relatively low participation rate in DanFunD of 34%. This raises concerns about the representativeness of the cohort, as differences between responders and non-responders could introduce potential bias. The results could be biased towards stronger associations since participants experiencing symptoms might be more prone to participate in a study with a specific focus on FSD. Conversely, the associations could also be weaker associations if individuals with the most disabling symptoms lacked the resources to participate. This issue was examined in a recent register-based non-responder analysis of the DanFunD cohort, which found that the prevalence of FSD and common mental disorders was higher among non-responders than among responders [8]. This suggests that non-responders might experience a higher symptom load than responders, and, consequently, the observed associations in this present study could be underestimated.

It is also a limitation that all parameters assessing lifestyle and mental health are based on self-reported measures in the questionnaires, as these results can potentially be affected by recall bias.

It could further be speculated that some symptoms used to identify cases with BDS could be explained by other conditions than BDS and that some BDS cases in the present study may be false positives. The influence of physical symptoms caused by somatic comorbidities on the prevalence estimates of FSD has been addressed in previous DanFunD studies. One study found that excluding participants with severe, chronic diseases from the analysis resulted in only a slight difference in prevalence estimates of BDS and functional somatic disorders, suggesting that the occurrence of somatic comorbidities might not substantially affect the overall prevalence of FSD [11].

Lastly, the relativity high amount of missing data on the step test used to estimate VO_2_-max could potentially bias these results. To complete the test efficiently enough to achieve a valid measure, a certain level of coordination, physical health, and endurance was required. This measure stands out according to the number of missing values, as almost 42% of all participants with multi-organ FSD and 26.5% of those with single-organ BDS could not complete the test. In comparison, only 11.5% of the controls could not complete the test. These missing data may have attenuated the observed associations because the most physically restricted individuals are most likely those who are not represented in the analysis.

## 7. Conclusions

We found an association between BDS and less optimal body composition, decreased cardiorespiratory health, and reduced physical performance in individuals with both single-organ and multi-organ BDS. Additionally, there was a tendency for individuals with multi-organ BDS to exhibit lower physical fitness or reduced cardiorespiratory health than those with single-organ BDS across all observed measures. This suggests a correlation between declining physical capacity and the presence of more widespread symptoms affecting multiple organ systems.

## Figures and Tables

**Table 1:** Characteristics of study participants. For BDS, characteristics are presented for the entire BDS sample as well as for the two BDS sub-groups, i.e., those with single-organ BDS and those with multi-organ BDS. Mean (SD) is presented unless otherwise stated. *p*-values presented correspond to the comparison between each of the three BDS subgroups and the non-BDS control population.

		Total BDS (n = 1544)	*p* ^a^	Single-Organ BDS (n = 1448)	*p* ^a^	Multi-Organ BDS (n = 96)	*p* ^a^	Non-BDS/Controls (n = 8035)
	Age, median (q1–q3)	55.0 (46–63)	0.062 ^Ŧ^	55.0 (47–63)	0.015 ^Ŧ^	51.0 (42–58)	0.029 ^Ŧ^	54.0 (44–64)
	Sex (% women)	67.5	<0.0001	66.8	<0.0001	77.1	<0.0001	51.3
Body Composition	Waist circumference							
	% Men > 102 cm ^b^	39.0	<0.0001	37.7	<0.0001	68.2	<0.0001	24.5
	% Women > 88 cm ^b^	40.5	<0.0001	39.6	<0.0001	51.4	<0.0001	28.0
	Fat Percentage							
	Men	26.3 (7.6)	<0.0001	26.1 (7.5)	<0.0001	29.1 (9.2)	0.0097	23.4 (6.5)
	Women	36.8 (7.9)	<0.0001	36.7 (7.8)	<0.0001	38.5 (8.7)	<0.0001	34.4 (7.5)
	Nutritional classification by BMI (kg/m^2^)		<0.0001		<0.0001		<0.0001	
	% Under + normal weight (BMI ≤ 24.99)	37.3		37.6		33.3		47.9
	% overweight (BMI ≤ 29.99), %	37.4		38.2		25.0		36.8
	% Obese (class I–III) (BMI ≥ 30.00), %	25.3		24.2		41.7		15.3
Cardiorespiratory health/physical performance	FVC (L)	3.8 (0.9)	<0.0001	3.8 (0.9)	<0.0001	3.6 (0.9)	<0.0001	4.1 (1.0)
	FEV1 (L)	2.9 (0.8)	<0.0001	2.9 (0.8)	<0.0001	2.8 (0.7)	<0.0001	3.15 (0.9)
	FEV1/FVC, median (q1–q3)	0.77 (0.72–0.81)	0.20 ^Ŧ^	0.77 (0.72–0.81)	0.22 ^Ŧ^	0.77 (0.71–0.80)	0.63 ^Ŧ^	0.77 (0.72–0.81)
	Systolic Blood Pressure (mmHg)	129.1 (18.4)	0.58	129.2 (18.5)	0.83	126.3 (15.9)	0.10	129.3 (18.1)
	Diastolic Blood Pressure (mmHg)	78.7 (10.2)	0.94	78.6 (10.2)	0.99	79.1 (9.8)	0.67	78.6 (10.3)
	Hand grip strength (kg)							
	Men, median (q1–q3)	43.09 (37.4–48.3)	<0.0001 ^Ŧ^	43.1 (37.5–48.4)	<0.0001 ^Ŧ^	40.1 (32.9–44.5)	0.02 ^Ŧ^	44.7 (39.2–50.4)
	Women, median (q1–q3)	25.4 (21.4–29.3)	<0.0001 ^Ŧ^	25.6 (21.6–29.5)	<0.0001 ^Ŧ^	24.0 (19.1–28.4)	<0.0001 ^Ŧ^	27.2 (23.8–31.3)
	VO_2_ max (mL/kg/min)	30.0 (9.0)	<0.0001	30.1 (9.1)	<0.0001	26.7 (7.5)	<0.0001	33.6 (9.5)
Lifestyle factors	Dietary Intake		<0.0001		<0.0001		0.0005	
	% Unhealthy	15.08		14.49		22.92		10.81
	% Middle	65.08		65.37		61.46		66.27
	% Healthy	19.84		20.14		15.63		22.92
	Smoking (% yes, daily or frequently)	21.57	<0.0001	21.57	<0.0001	21.88	0.08	15.42
	Alcohol, drinks/week							
	Men, median (q1–q3)	8.5 (3.0–17.0)	0.30 ^Ŧ^	9.0 (3.0–17.0)	0.13 ^Ŧ^	3.5 (1.0–6.0)	0.01 ^Ŧ^	7.75 (3.5–14.5)
	Women, median (q1–q3)	3.0 (1.0–7.0)	0.0002 ^Ŧ^	3.0 (1.0–7.0)	0.007 ^Ŧ^	2.0 (0.0–4.0)	<0.0001 ^Ŧ^	4.0 (1.0–7.5)
	Physical activity (% low and very low)	79.1	<0.0001	78.5	<0.0001	88.5	<0.0001	66.6
	Waking up early (% yes, daily/almost daily)	53.45	<0.0001	52.05	<0.0001	73.96	<0.0001	33.04
	Cannot sleep (% yes, daily/almost daily)	37.42	<0.0001	35.81	<0.0001	62.11	<0.0001	14.32
Mental health factors	Cohens Stress Scale, median (q1–q3)	14.0 (9.0–19.0)	<0.0001 ^Ŧ^	14.0 (9.0–19.0)	<0.0001 ^Ŧ^	20.0 (15.0–24.0)	<0.0001 ^Ŧ^	9.0 (5.0–13.0)
	SCL Anxiety, median (q1–q3)	4.0 (1.0–8.0)	<0.0001 ^Ŧ^	4.0 (1.0–7.0)	<0.0001 ^Ŧ^	12.5 (8.0–17.0)	<0.0001 ^Ŧ^	1.0 (0.0–3.0)
	SCL Depression, median (q1–q3)	8.0 (4.0–14.0)	<0.0001 ^Ŧ^	7.0 (3.0–13.0)	<0.0001 ^Ŧ^	18.0 (10.0–27.0)	<0.0001 ^Ŧ^	2.0 (1.0–5.0)

^a^ Tested mean/median differences between BDS and non-BDS using Kruskal-Wallis test for skewed variables ^Ŧ^ otherwise *t*-test. Chi-squared was applied for categorical variables. ^b^ Adapted based on recommended cutoff from WHO [25]. FVC Forced Vital Capacity, FEV1 Forced Expiratory Volume first second, VO_2_-max Maximal Oxygen Uptake, q1–q3 25 and 75 percentiles.

**Table 2:** Association between body composition and BDS case status compared to controls.

	Regression Coefficient [95% CI]		
	Model 1	Model 2	Model 3
Controls	0.00	0.00	0.00
Waist circumference (cm)			
Total BDS	**1.048 [1.040; 1.055]**	**1.039 [1.032; 1.047]**	**1.038 [1.030; 1.046]**
Single-organ BDS	**1.044 [1.036; 1.051]**	**1.037 [1.030; 1.045]**	**1.037 [1.029; 1.045]**
Multi-organ BDS	**1.109 [1.081; 1.137]**	**1.072 [1.044; 1.102]**	**1.072 [1.042; 1.104]**
Fat percentage (%)			
Total BDS	**2.440 [2.064; 2.816]**	**2.065 [1.670; 2.459]**	**2.005 [1.588; 2.423]**
Single-organ BDS	**2.282 [1.897; 2.667]**	**2.009 [1.608; 2.411]**	**1.972 [1.551; 2.394]**
Multi-organ BDS	**4.729 [3.361; 6.096]**	**3.156 [1.692; 4.619]**	**3.438 [1.875; 5.000]**
BMI (kg/m^2^)			
Total BDS	**1.058 [1.049; 1.068]**	**1.051 [1.041; 1.061]**	**1.049 [1.038; 1.059]**
Single-organ BDS	**1.054 [1.044; 1.633]**	**1.049 [1.039; 1.059]**	**1.047 [1.037; 1.058]**
Multi-organ BDS	**1.128 [1.093; 1.165]**	**1.086 [1.049; 1.124]**	**1.092 [1.053; 1.133]**

Model 1: Adjusted for sex and age. Model 2: Additionally adjusted for dietary intake, smoking, alcohol intake, physical activity, and sleep disturbances. Model 3: Additionally adjusted for self-perceived stress, anxiety, and depression. Bold = significant on a 5% significance level.

**Table 3:** Association between cardiorespiratory health/physical performance and BDS case status compared to controls.

	Regression Coefficient [95% CI]		
	Model 1	Model 2	Model 3
Controls	0.00	0.00	0.00
FVC (L) ^1^			
Total BDS	**−0.057 [−0.089; −0.026]**	**−0.037 [−0.071; −0.003]**	**−0.039 [−0.075; −0.003]**
Single-organ BDS	**−0.054 [−0.087; −0.021]**	**−0.035 [−0.070; −0.001]**	−0.036 [−0.072; 0.001]
Multi-organ BDS	−0.107 [−0.225; 0.010]	−0.072 [−0.200; 0.056]	−0.093 [−0.230; 0.043]
FEV1 (L) ^1^			
Total BDS	**−0.058 [−0.873; −0.028]**	−0.028 [−0.060: 0.003]	−0.024 [−0.058; 0.009]
Single-organ BDS	**−0.054 [−0.084; −0.024]**	−0.025 [−0.058; 0.007]	−0.021 [−0.054; 0.013]
Multi-organ BDS	**−0.123 [−0.233; −0.012]**	−0.089 [−0.209; 0.031]	−0.096 [−0.223; 0.032]
FEV1/FVC			
Total BDS	−0.002 [−0.008; 0.003]	−0.000 [−0.006; 0.005]	0.001 [−0.005; 0.007]
Single-organ BDS	−0.002 [−0.007; 0.004]	0.000 [−0.006; 0.006]	0.002 [−0.005; 0.008]
Multi-organ BDS	−0.011 [−0.031; 0.009]	−0.012 [−0.035; 0.010]	−0.011 [−0.035; 0.013]
Systolic BP (mmHg)			
Total BDS	0.999 [0.993; 1.006]	1.002 [0.994; 1.009]	1.005 [0.997; 1.012]
Single-organ BDS	1.000 [0.993; 1.006]	1.002 [0.995; 1.010]	1.005 [0.998; 1.013]
Multi-organ BDS	0.995 [0.971; 1.020]	0.988 [0.962; 1.014]	0.989 [0.961; 1.017]
Diastolic BP (mmHg)			
Total BDS	1.006 [0.999; 1.013]	1.006 [0.999; 1.014]	1.007 [1.000; 1.015]
Single-organ BDS	1.005 [0.998; 1.012]	1.006 [0.998; 1.013]	1.007 [0.999; 1.015]
Multi-organ BDS	1.024 [0.999; 1.050]	1.015 [0.998; 1.043]	1.016 [0.987; 1.046]
Handgrip strength (kg)			
Total BDS	**−1.864 [−2.233; −1.486]**	**−1.471 [−1.866; −1.077]**	**−1.306 [−1.722; −0.889]**
Single-organ BDS	**−1.687 [−2.064; −1.310]**	**−1.349 [−1.751; −0.948]**	**−1.195 [−1.616; −0.774]**
Multi-organ BDS	**−4.580 [−5.936; −3.224]**	**−3.638 [−5.117; −2.159]**	**−3.811 [−5.385; −2.238]**
VO_2_ max (mL/kg/min)			
Total BDS	**−3.339 [−3.865; −2.814]**	**−2.292 [−2.825; −1.759]**	**−2.143 [−2.707; −1.579]**
Single-organ BDS	**−3.171 [−3.708; −2.634]**	**−2.180 [−2.723; −1.637]**	**−2.053 [−2.623; −1.482]**
Multi-organ BDS	**−6.551 [−8.734; −4.368]**	**−4.747 [−6.945; −2.549]**	**−5.100 [−7.420; −2.780]**

Model 1: Adjusted for sex and age. Model 2: Additionally adjusted for dietary intake, smoking, alcohol intake, physical activity, and sleep disturbances. Model 3: Additionally adjusted for self-perceived stress, anxiety, and depression. ^1^ FVC and FEV1 also adjusted for height and weight in all models. Bold = significant on a 5% significance level.

## Data Availability

The datasets used and analyzed during the current study are not publicly available for ethical and legal reasons. Public availability may compromise participant privacy, and this would not comply with Danish legislation. Data are available from the corresponding author upon reasonable request. Acquisition of data is only allowed after permission to handle data has been obtained in accordance with the guidelines stated by the Danish Data Protection Agency: http://www.datatilsynet.dk/english (accessed on 1 September 2024). Access to data included in this study can be gained through submitting a request to the corresponding author and will have to be approved by the Capital Region Knowledge Center for Data Compliance, Capital Region Denmark; cru-fp-vfd@regionh.dk.

## Abbreviations

DanFunD: Danish Study of Functional Disorders; BDS: bodily distress syndrome; FSS: functional somatic syndromes; cm: centimeter; BMI: Body mass index; FVC: Forced vital performance; FEV: Forced expiratory volume; VO_2_-max: Maximal oxygen consumption; ICD: International Statistical Classification of Diseases and Related Health Problems; FFQ: Food frequency questionnaire; CI: confidence intervals.
